# Supplementary material for: Physiological and Metabolic Responses of Rice to Reduced Soil Moisture: Relationship of Water Stress Tolerance and Grain Production
Source: Int J Mol Sci. 2019 Apr 15;20(8):1846. doi: 10.3390/ijms20081846 (PMC6514846; doi:10.3390/ijms20081846)
Supplement: Supplementary file 1 [file ijms-20-01846-s001.pdf]

International Journal of
Molecular Sciences

MDPI

Article

Jinyoung Y. Barnaby [1], Jai S. Rohila [1], Chris G. Henry [2], Richard C. Sicher [3], Vagimalla R. Reddy [3] and Anna M. McClung [1,*]

[1] USDA-ARS Dale Bumpers National Rice Research Center, Stuttgart, AR 72160, USA; jinyoung.barnaby@ars.usda.gov (J.Y.B.); jai.rohila@ars.usda.gov (J.S.R.)
[2] Department of Biological and Agricultural Engineering, University of Arkansas, Fayetteville, AR 72701, USA; cghenry@uark.edu
[3] USDA-ARS Adaptive Cropping Systems Laboratory, Henry A. Wallace Beltsville Agricultural Research Center, Beltsville, MD 20705, USA; rsicher1981@gmail.com (R.C.S.); vangimalla.reddy@ars.usda.gov (V.R.R.)
[*] Correspondence: Anna.Mcclung@ars.usda.gov; Tel.: +1-870-672-9300; Fax: +1-870-673-7581

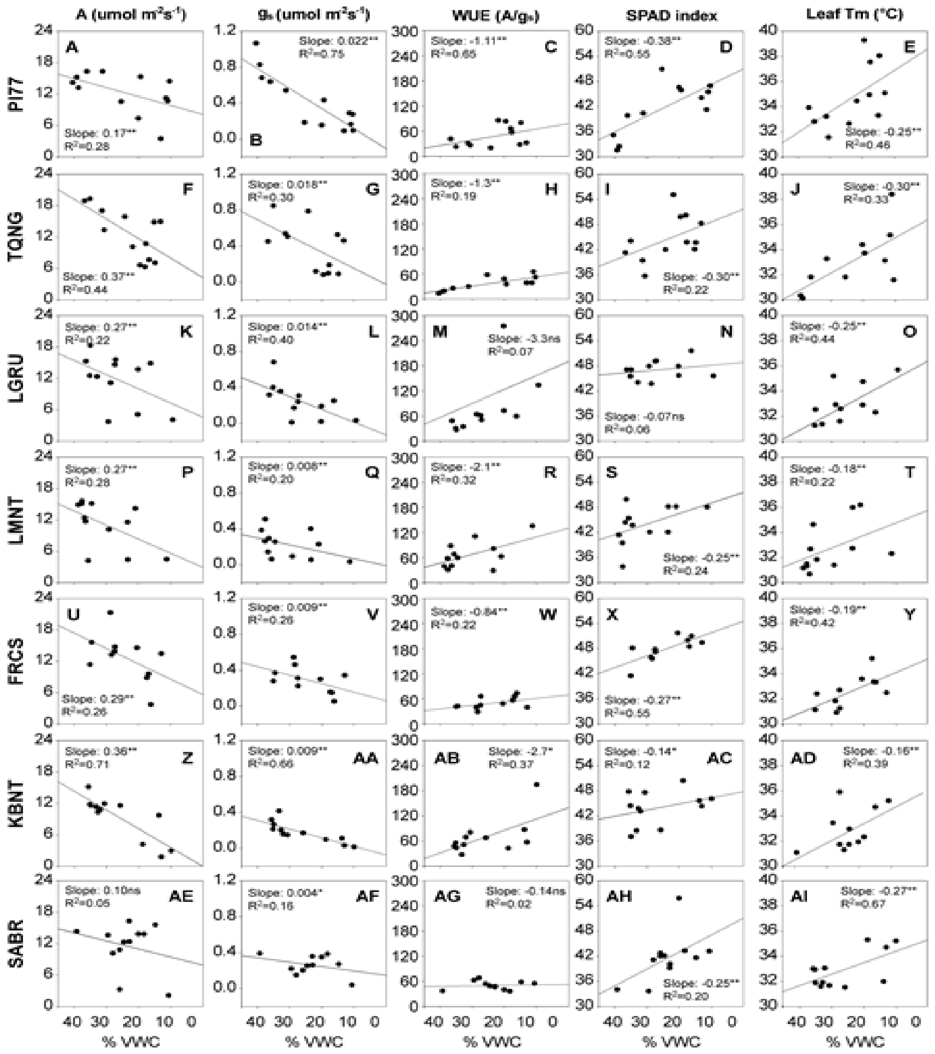

Figure S1. Physiological responses to different levels of irrigation as measured by volumetric water content (horizontal axis) of seven cultivars. The y-axis shows values of A, g_s, WUE (A/ g_s), SPAD index, and leaf Tm. Other details are as in Figure 1.

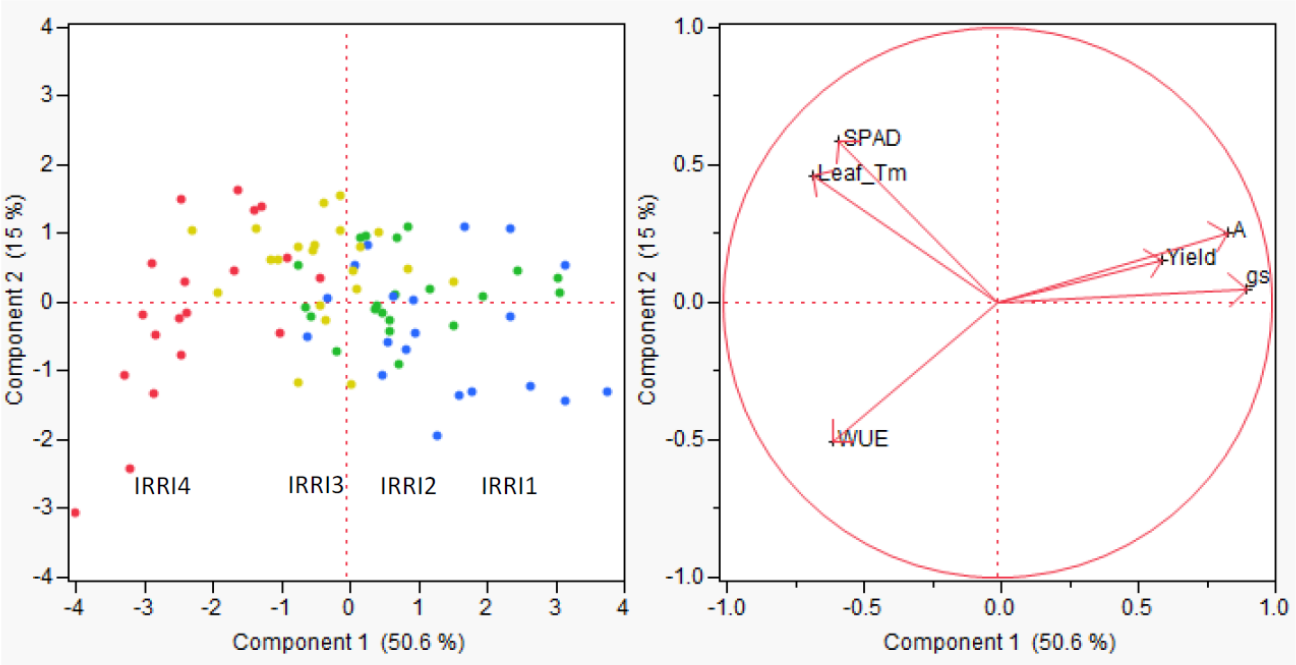

Figure S2. Principal component analysis of physiological parameters, A, g_s, WUE (A/ g_s), SPAD index, and leaf Tm among seven cultivars in response to four different levels of water stress (IRRI_1 to 4) (A), and eigenvector values of PC1 and PC2. Blue, green, yellow, and red dots represent IRRI_1, 2, 3, and 4, respectively.

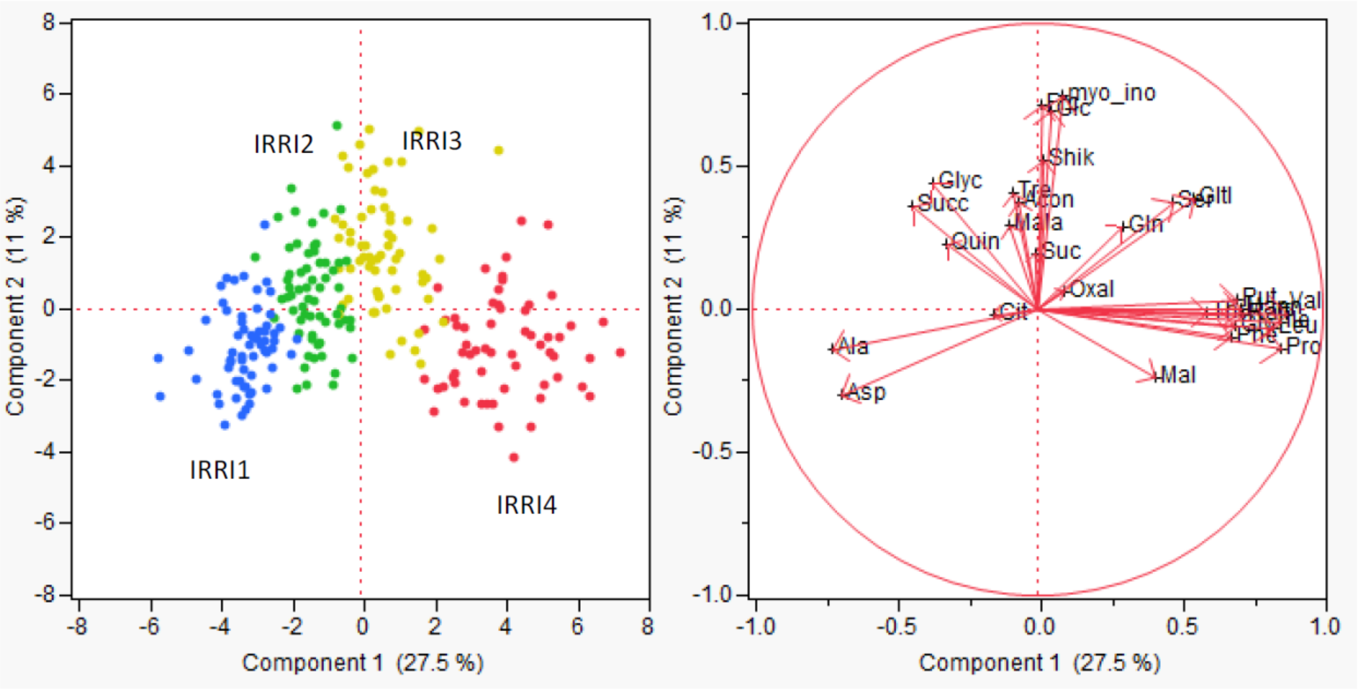

Figure S3. Principal component analysis of 26 metabolites among seven cultivars in response to four different levels of water stress (IRRI_1 to 4) (A), and eigenvector values of PC1 and PC2. Other details are as in Figure S2.

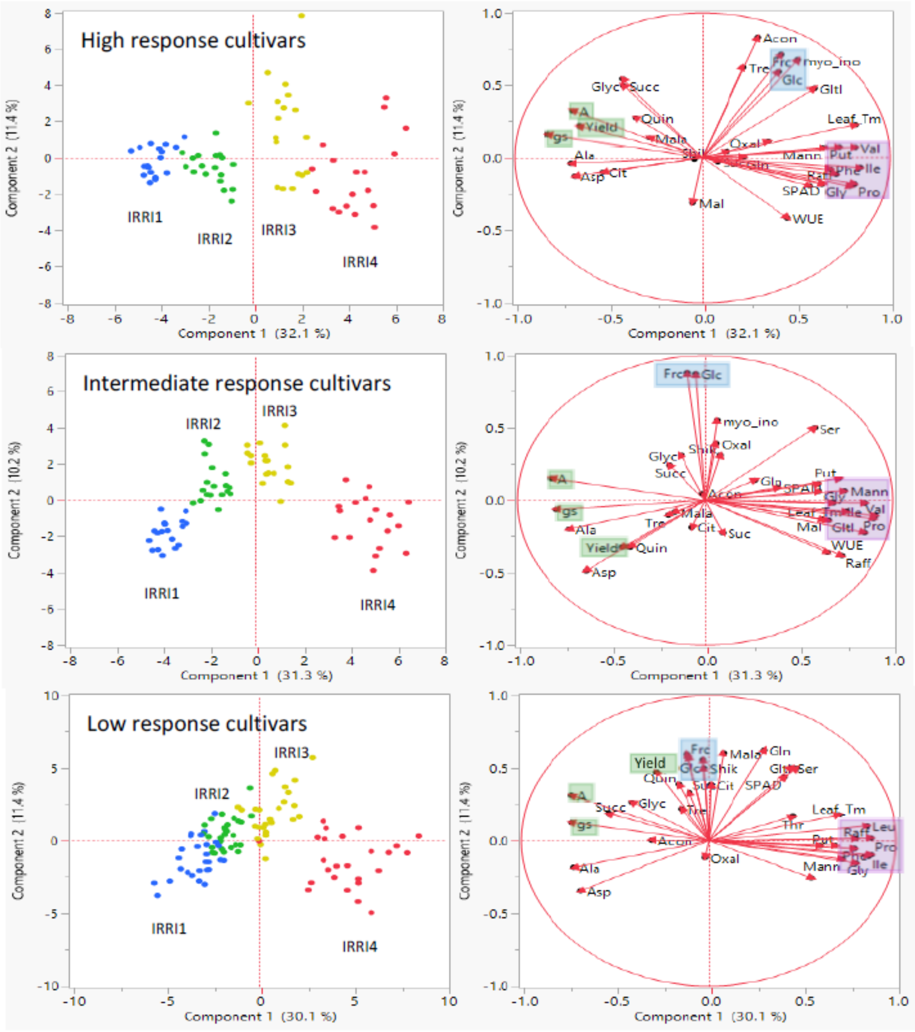

Figure S4. Principal component analysis of physiological and metabolic traits among seven cultivars in response to four different levels of water stress (IRRI_1 to 4) (A), and eigenvector values of PC1 and PC2. Other details are as in Figure S2.

Table S1A. Means and SE of yield (grain weight per plant (g)) of 7 rice cultivars under 4 different irrigation levels (IRRI_1 to 4).

Yield response category	Cultivars	Year	IRRI_1		IRRI_2		IRRI_3		IRRI_4	
			Mean	SE	Mean	SE	Mean	SE	Mean	SE
High response	TQNG	2014	38.77	4.97	36.97	5.91	26.43	4.10	3.27	1.48
		2015	55.80	8.05	61.57	5.32	NA	NA	20.90	9.88
		2016	47.00	2.78	56.00	4.91	36.67	12.48	28.50	0.58
	PI77	2014	31.60	6.13	13.47	2.69	16.90	8.07	3.73	2.14
		2015	37.30	8.99	28.47	5.82	NA	NA	20.83	2.40
		2016	47.00	3.51	36.33	7.49	26.33	5.67	20.67	5.49
Intermediate response	LGRU	2014	25.00	1.67	19.60	4.10	18.90	0.15	7.80	4.18
		2015	13.60	0.40	19.80	4.40	NA	NA	13.15	2.45
		2016	26.50	1.04	18.83	4.41	13.50	5.62	9.33	4.48
	LMNT	2014	16.93	0.73	16.70	4.27	9.97	3.78	4.93	3.10
		2015	15.43	3.91	6.20	NA	NA	NA	13.23	9.23
		2016	17.33	2.77	19.17	1.17	16.83	4.21	4.03	2.15
Non- or low response	FRCS	2014	38.03	1.68	17.60	8.74	27.00	12.16	20.60	7.55
		2015	31.13	5.40	48.00	4.10	NA	NA	26.80	3.55
		2016	37.67	6.89	34.83	0.93	46.00	5.84	29.33	3.71
	KBNT	2014	16.03	6.53	10.73	4.71	18.47	10.09	4.57	1.73
		2015	13.10	3.41	8.95	1.75	NA	NA	5.13	1.68
		2016	14.17	5.13	16.17	2.46	19.33	2.80	15.00	4.54
	SABR	2014	30.63	3.54	31.67	3.02	23.20	6.94	20.93	4.06
		2015	19.00	3.06	23.97	2.09	NA	NA	18.43	2.07
		2016	24.50	0.76	24.33	1.74	23.33	4.85	21.17	2.35

Table S1. Analysis of variance of seven cultivars (C) evaluated across four irrigation levels (I) in each of three years for grain weight per plant (g). ** and ns indicate $p < 0.01$ and $p > 0.01$, respectively.

Year	Source of Variation	Prob. > F
2014	Cultivar (C)	**
	Irrigation (I)	**
	C x I	ns
2015	Cultivar (C)	**
	Irrigation (I)	**
	C x I	ns
2016	Cultivar (C)	**
	Irrigation (I)	**
	C x I	ns

Table S2. Average daily air temperature, soil temperature and total daily solar radiation during vegetative stage (DOE 1 to 103) and reproductive stage (DOE 104 to 154) at Stuttgart, Arkansas from 2014 to 2016. The stage of vegetation/reproduction and grain fill was decided based on heading date in 2016 (Suppl. Table 9).

	Average air temperature (°C)			Average soil temperature (°C)			Total Solar flux (MJ/m²)		
	2014	2015	2016	2014	2015	2016	2014	2015	2016
Vegetative/reproductive stage (DOE 1-103)	25.3	21.4	27.8	25.8	22.7	27.7	27.2	23.8	29.1
Grain fill stage (DOE 104-154)	20.8	17.0	22.9	25.0	17.7	26.8	18.6	13.6	21.4

Table 3. Summary of amount of irrigation applied for IRRI 1 to 4 in each of three years.

Treatment		Water applied (cm ha^{-1})		
		2014	2015	2016
IRRI1	30% VWC	40.64	78.97	87.33
IRRI2	24% VWC	44.63	59.03	75.64
IRRI3	20% VWC	37.92	57.02	62.97
IRRI4	14% VWC	25.30	40.13	43.59

Table S4A. Mean and SE of physiological traits of 7 rice cultivars under 4 different irrigation levels (IRRI_1 to 4) in 2016.

Cultivars	Traits	IRRI_1		IRRI_2		IRRI_3		IRRI_4	
		Mean	SE	Mean	SE	Mean	SE	Mean	SE
FRCS	A	15.50	1.48	14.06	0.38	12.93	0.84	6.27	1.14
	Gs	0.38	0.04	0.39	0.02	0.22	0.02	0.11	0.02
	WUE (A/g_s)	41.65	0.81	36.66	2.07	59.65	2.83	64.97	3.73
	SPAD	45.07	0.93	47.70	0.56	49.13	0.67	50.50	0.22
	Leaf Tm	89.43	0.41	89.53	0.46	90.97	0.67	93.70	0.76
	Seed weight per plant (g)	37.67	6.89	34.83	0.93	46.00	5.84	29.33	3.71
KBNT	A	11.25	0.24	11.34	0.17	10.30	1.62	4.82	1.23
	Gs	0.23	0.01	0.24	0.04	0.20	0.03	0.05	0.01
	WUE (A/g_s)	50.14	1.75	59.11	8.14	52.84	3.83	112.99	20.83
	SPAD	41.77	1.17	43.07	1.31	45.60	1.79	45.33	0.27
	Leaf Tm	89.87	0.31	89.60	0.43	91.73	0.99	92.97	0.90
	Seed weight per plant (g)	14.17	5.13	16.17	2.46	19.33	2.80	15.00	4.54
LGRU	A	15.15	0.84	14.40	0.52	13.24	0.55	4.32	0.21
	Gs	0.44	0.06	0.33	0.01	0.20	0.01	0.02	0.00
	WUE (A/g_s)	39.81	5.38	44.68	2.52	65.47	1.97	258.41	33.87
	SPAD	47.23	0.52	46.77	0.76	47.00	1.19	47.13	0.38
	Leaf Tm	89.27	0.34	88.20	0.77	92.03	0.66	94.30	0.79
	Seed weight per plant (g)	26.50	1.04	18.83	4.41	13.50	5.62	9.33	4.48
LMNT	A	15.20	0.11	13.04	0.54	8.69	1.15	7.68	1.63
	Gs	0.38	0.04	0.27	0.04	0.15	0.04	0.10	0.03
	WUE (A/g_s)	42.39	3.99	58.58	8.48	72.73	10.27	92.90	10.89
	SPAD	38.23	1.14	43.40	0.34	45.80	1.13	48.17	0.02
	Leaf Tm	88.33	0.07	89.10	0.53	91.17	0.86	94.63	1.13
	Seed weight per plant (g)	17.33	2.77	19.17	1.17	16.83	4.21	4.03	2.15
PI77	A	14.20	0.29	14.06	0.89	12.71	1.36	8.47	1.24
	Gs	0.86	0.06	0.42	0.07	0.32	0.06	0.15	0.04
	WUE (A/g_s)	17.02	0.94	39.62	4.78	43.61	3.44	160.40	54.22
	SPAD	33.10	0.54	45.63	1.60	44.67	1.07	43.63	0.62
	Leaf Tm	86.30	0.09	90.33	0.57	91.47	0.73	95.97	1.38
	Seed weight per plant (g)	47.00	3.51	36.33	7.49	26.33	5.67	20.67	5.49
SABR	A	13.44	0.28	14.13	0.59	13.20	0.81	5.38	1.37

	Gs	0.29	0.03	0.33	0.02	0.26	0.03	0.12	0.04
	WUE (A/g_s)	48.83	3.71	43.39	1.91	55.07	4.14	53.92	0.20
	SPAD	35.90	1.05	41.53	0.61	46.17	2.41	42.63	0.17
	Leaf Tm	88.33	0.80	89.13	0.26	91.97	0.72	93.67	1.16
	Seed weight per plant (g)	24.50	0.76	24.33	1.74	23.33	4.85	21.17	2.35
**TQNG**	A	16.57	0.87	16.54	0.61	11.95	0.76	6.67	0.11
	Gs	0.63	0.05	0.59	0.05	0.26	0.05	0.06	0.01
	WUE (A/g_s)	26.89	1.27	30.04	3.14	58.55	7.72	274.31	96.34
	SPAD	39.73	1.22	41.80	0.13	49.07	1.64	47.90	1.05
	Leaf Tm	86.90	0.44	88.27	0.33	92.60	0.84	95.77	1.16
	Seed weight per plant (g)	47.00	2.78	56.00	4.91	36.67	12.48	28.50	0.58

Table S4B. Summary ANOVAs and C, I, C x I effects for five physiological traits evaluated at four irrigation (I) levels of seven varieties (C) during 2016. ** indicates $p < 0.01$.

	**Cultivar (C)**	**Irrigation (I)**	**C x I**
A	**	**	**
Gs	**	**	**
WUE (A/g_s)	**	**	**
Leaf Tm	ns	**	**
SPAD	**	**	**
Seed Weight	**	**	*

Table S5A. Means and SE of metabolites of 7 rice cultivars under 4 different irrigation levels (IRRI_1 to 4) during 2016.

**Cultivars**	**Metabolite**	**IRRI 1**		**IRRI 2**		**IRRI 3**		**IRRI 4**	
		**Mean**	**SE**	**Mean**	**SE**	**Mean**	**SE**	**Mean**	**SE**
**FRCS**	**Val**	18.07	2.23	54.18	6.19	84.76	8.29	156.17	19.54
	**Phe**	10.27	1.03	28.49	3.10	38.31	7.83	103.13	14.28
	**Ser**	188.97	15.46	261.27	32.84	564.36	51.31	489.48	83.43
	**Gln**	3694.49	1013.19	4037.87	630.12	8902.52	1214.89	6563.92	563.87
	**Ala**	762.80	54.89	766.54	60.07	506.08	59.01	291.75	32.08
	**Asp**	1311.58	94.35	655.58	51.48	282.99	37.88	108.08	11.05

	**Pro**	9.23	0.02	12.11	1.55	43.99	1.73	88.85	10.44
	**Thr**	77.67	9.13	198.33	49.87	214.30	62.71	283.15	74.21
	**Frc**	4344.14	645.19	10842.47	1035.88	10311.68	1061.73	5286.02	521.39
	**Glc**	3758.99	591.89	11144.64	1590.30	12367.92	1313.53	5025.83	467.88
	**myo-ino**	387.00	80.20	727.59	65.34	833.30	106.39	549.92	55.74
	**Gltl**	23.50	4.09	68.50	16.18	62.87	15.57	85.82	12.67
	**Suc**	57873.13	3423.69	74035.12	2664.20	72152.64	2502.75	56110.88	3304.07
	**Mann**	9.21	0.03	10.20	1.06	20.24	5.17	34.20	4.43
	**Tre**	30.66	5.91	34.81	4.10	27.41	5.25	38.25	6.78
	**Raff**	247.80	32.66	470.06	45.42	1226.39	140.23	3089.25	478.71
	**Mal**	22.54	2.24	19.37	2.82	23.53	1.60	49.77	5.74
	**Succ**	47.05	3.67	46.86	6.59	52.71	4.62	39.79	5.62
	**Cit**	481.04	201.79	263.22	120.93	880.94	235.28	735.78	228.87
	**Oxal**	154.33	32.26	431.00	157.87	278.88	109.49	446.18	110.37
	**Glyc**	262.16	37.64	269.55	42.88	414.33	51.42	201.22	24.74
	**Mala**	555.04	209.97	382.57	110.54	1872.35	254.64	771.46	287.42
	**Acon**	207.19	24.86	164.75	27.87	57.99	7.73	47.85	12.08
	**Quin**	125.75	35.98	108.15	34.12	203.66	66.57	61.67	7.41
	**Shik**	207.00	30.61	224.43	74.13	404.49	77.07	169.82	28.70
	**Put**	12.12	1.56	19.49	2.46	22.31	2.17	32.65	2.04
**KBNT**	**Val**	13.36	2.24	29.79	3.34	70.78	5.04	114.03	8.02
	**Phe**	9.28	0.01	19.37	3.25	46.66	4.18	145.03	32.68
	**Ser**	236.39	25.89	443.62	69.98	317.68	44.24	363.32	84.73
	**Gln**	4001.43	514.92	4817.82	422.51	6634.58	740.58	4472.39	1035.13
	**Ala**	788.13	107.57	495.34	58.99	397.82	39.05	232.41	31.08
	**Asp**	1590.16	172.85	513.62	39.54	213.76	35.25	175.96	31.11
	**Pro**	8.47	0.81	20.32	2.49	34.18	3.43	72.14	9.78
	**Thr**	98.26	14.94	137.04	29.21	108.00	13.58	151.32	17.89

	**Frc**	2736.39	503.35	6964.94	568.55	7319.36	719.02	3637.07	409.66
	**Glc**	2724.64	399.09	10991.46	4291.00	8968.89	821.42	4065.20	569.77
	**myo-ino**	609.21	118.11	837.36	70.35	832.40	57.37	407.32	85.78
	**Gltl**	46.48	18.97	45.42	9.97	81.52	16.99	73.61	15.16
	**Suc**	66965.93	4319.29	63609.42	3061.11	66844.36	3297.09	62377.41	4504.40
	**Mann**	10.34	1.03	13.42	1.62	20.57	2.06	39.27	5.30
	**Tre**	26.89	2.44	42.36	4.16	38.71	4.76	18.41	3.13
	**Raff**	170.16	12.12	437.97	72.32	2016.40	522.90	3303.93	279.19
	**Mal**	12.39	2.19	18.64	2.21	15.32	1.53	35.81	6.83
	**Succ**	66.16	4.26	77.55	10.65	47.85	4.58	22.52	3.16
	**Cit**	227.98	57.89	568.03	145.58	290.06	78.42	204.30	52.82
	**Oxal**	195.54	40.35	476.54	196.86	453.01	99.33	331.89	32.89
	**Glyc**	469.27	43.40	552.23	81.12	381.32	82.99	186.42	24.69
	**Mala**	1255.14	339.00	754.54	163.37	1196.38	244.96	583.44	257.69
	**Acon**	115.71	26.28	93.70	11.07	130.17	17.97	119.06	29.90
	**Quin**	311.65	77.24	164.63	36.64	395.82	139.81	33.81	9.41
	**Shik**	220.30	38.30	235.48	57.92	527.17	81.00	168.28	22.80
	**Put**	13.37	1.66	13.36	2.25	21.71	1.57	33.04	2.81
**LGRU**	**Val**	21.53	4.61	28.43	4.04	51.43	2.76	130.04	15.59
	**Phe**	9.26	0.02	9.36	0.02	60.58	14.78	97.04	30.39
	**Ser**	153.61	11.34	230.26	30.11	562.09	41.11	399.32	61.27
	**Gln**	1785.48	299.20	4232.98	423.16	6171.72	1127.71	6555.10	760.52
	**Ala**	905.02	75.37	503.27	27.75	573.31	49.42	265.73	33.94
	**Asp**	1781.12	295.31	356.71	42.66	200.44	36.36	163.02	28.39
	**Pro**	9.30	0.03	13.47	1.62	45.48	5.39	89.80	5.53
	**Thr**	58.30	14.22	73.00	20.92	198.06	34.20	248.46	55.11
	**Frc**	2789.82	410.41	10111.79	1670.41	11362.23	965.35	2394.74	411.07
	**Glc**	2646.02	522.51	12154.40	2100.81	13334.64	976.60	2834.54	420.57

	**myo-ino**	417.97	90.43	628.87	81.71	740.61	93.41	423.41	65.72
	**Gltl**	17.47	3.63	19.49	2.89	24.67	3.41	93.18	24.03
	**Suc**	65927.88	2809.36	61772.83	2700.26	56661.61	3716.80	67092.01	3855.31
	**Mann**	12.33	1.54	15.43	2.21	23.90	3.52	36.46	4.46
	**Tre**	49.46	8.49	33.80	5.72	29.93	6.21	30.69	4.73
	**Raff**	300.17	11.35	323.37	28.28	379.18	44.79	4188.19	700.27
	**Mal**	12.38	2.26	18.70	2.78	21.70	2.29	43.77	4.48
	**Succ**	34.87	6.24	63.53	5.95	54.53	6.53	50.98	8.85
	**Cit**	331.64	107.35	635.46	73.61	274.92	44.90	557.33	125.32
	**Oxal**	190.21	35.29	630.59	257.26	581.89	190.77	307.59	61.43
	**Glyc**	286.14	41.29	355.62	45.44	501.12	77.21	322.88	73.13
	**Mala**	792.60	290.30	821.28	100.73	1070.39	186.71	935.36	228.52
	**Acon**	140.45	41.79	117.23	16.65	35.93	9.79	84.17	12.48
	**Quin**	724.37	206.01	80.98	10.16	122.35	36.88	83.71	24.51
	**Shik**	166.16	30.59	185.40	20.78	220.15	44.08	215.16	43.72
	**Put**	10.31	1.06	16.57	3.05	21.78	4.40	29.04	4.55
**LMNT**	**Val**	17.34	2.37	30.66	4.95	75.04	5.17	159.99	11.60
	**Phe**	9.23	0.02	13.16	1.60	27.23	2.63	75.32	16.36
	**Ser**	202.48	26.16	309.21	40.30	462.67	42.04	444.51	44.28
	**Gln**	6110.24	1027.15	5584.34	576.96	5716.14	937.43	4846.67	639.37
	**Ala**	987.61	126.17	660.02	54.47	425.34	44.43	348.57	49.32
	**Asp**	1968.46	232.61	384.77	66.08	157.34	15.79	159.86	33.67
	**Pro**	14.30	3.53	21.71	3.48	37.36	2.24	86.79	11.23
	**Thr**	117.41	44.13	115.24	16.20	240.46	41.83	273.54	43.95
	**Frc**	2895.71	368.78	5753.56	721.56	8733.07	770.04	4226.47	795.38
	**Glc**	3170.41	371.15	5703.28	771.30	10311.91	1451.26	4909.32	897.93
	**myo-ino**	502.93	57.21	842.08	61.95	881.26	119.80	698.24	63.47
	**Gltl**	32.97	6.39	45.86	8.71	71.78	9.00	107.52	11.77

	**Suc**	63218.04	2702.43	60020.26	3278.24	69092.00	4156.21	65074.44	4359.91
	**Mann**	11.36	1.39	28.89	3.83	26.86	2.48	42.74	4.52
	**Tre**	36.94	4.88	40.81	7.16	42.88	9.65	31.71	2.68
	**Raff**	481.17	40.87	575.51	51.84	1429.83	202.45	2726.70	572.32
	**Mal**	14.27	1.63	17.41	1.86	18.41	2.19	23.69	3.46
	**Succ**	63.87	4.22	74.42	12.40	53.15	6.71	39.95	7.01
	**Cit**	566.15	205.06	485.87	76.64	333.64	62.82	317.38	71.55
	**Oxal**	259.85	99.15	763.27	305.36	726.71	209.97	407.79	116.05
	**Glyc**	416.67	48.54	385.63	59.96	427.43	83.16	254.79	54.45
	**Mala**	1486.04	378.89	1137.60	212.69	747.14	144.83	718.10	209.05
	**Acon**	96.74	19.40	142.22	31.03	148.83	38.63	155.51	36.37
	**Quin**	336.04	85.65	139.88	32.45	253.63	40.00	102.19	39.09
	**Shik**	255.79	54.80	343.49	59.53	603.02	71.29	267.79	83.52
	**Put**	11.29	1.34	15.30	2.20	30.96	2.19	44.69	4.91
**PI77**	**Val**	17.20	2.38	30.11	4.23	91.36	10.85	124.82	20.57
	**Phe**	11.33	1.39	17.50	2.43	59.01	15.34	127.96	5.98
	**Ser**	243.02	46.30	301.38	30.34	426.61	65.83	346.86	52.55
	**Gln**	3920.24	773.49	6030.20	788.29	6534.31	541.83	4342.12	437.83
	**Ala**	722.16	52.90	528.00	47.73	355.67	45.03	262.13	29.12
	**Asp**	1551.57	115.27	619.80	85.19	324.37	55.27	194.66	32.03
	**Pro**	12.40	1.55	20.70	2.08	27.91	2.17	90.83	10.05
	**Thr**	86.57	25.18	123.22	21.66	195.96	42.32	317.94	76.35
	**Frc**	2216.07	388.42	2692.67	194.99	6916.41	1688.08	4652.79	584.46
	**Glc**	2335.77	295.25	2970.37	227.20	7155.20	1778.23	4357.96	457.07
	**myo-ino**	215.13	32.06	340.43	50.42	728.97	114.01	542.57	106.86
	**Gltl**	24.78	5.84	12.37	1.56	65.69	15.19	82.69	23.49
	**Suc**	58987.82	3891.80	65382.66	4773.03	67060.22	3921.35	59584.06	1411.42
	**Mann**	14.76	1.76	18.58	2.70	24.18	3.42	44.73	4.59

	**Tre**	24.71	2.64	21.48	3.94	41.82	8.56	35.86	5.80
	**Raff**	233.37	36.65	400.88	59.43	727.13	77.55	2267.04	313.50
	**Mal**	31.29	4.09	32.91	4.40	16.51	2.09	21.89	6.10
	**Succ**	67.08	8.37	47.23	3.52	58.51	7.52	35.74	5.29
	**Cit**	1145.01	175.11	536.79	86.40	347.27	33.60	95.51	13.48
	**Oxal**	545.90	198.95	299.38	176.02	770.56	222.23	379.67	81.78
	**Glyc**	371.98	72.32	431.65	55.57	419.26	58.35	213.15	21.65
	**Mala**	1526.20	173.27	841.12	280.41	1029.74	279.22	105.31	11.43
	**Acon**	76.23	8.53	52.22	11.49	164.26	43.47	148.00	39.22
	**Quin**	322.94	79.90	70.12	44.80	118.03	52.41	39.91	5.87
	**Shik**	245.56	43.35	394.67	34.47	250.19	45.34	128.62	13.04
	**Put**	10.23	0.97	15.57	3.13	24.13	2.26	39.48	4.45
**SABR**	**Val**	14.38	2.28	23.77	4.44	40.83	4.90	94.76	11.07
	**Phe**	9.24	0.03	21.62	2.67	71.07	15.75	87.94	14.07
	**Ser**	167.24	21.09	313.28	44.94	295.97	53.28	280.63	26.77
	**Gln**	3396.18	723.62	4969.00	477.72	6848.40	1261.85	4573.56	440.96
	**Ala**	1078.78	123.03	612.86	31.15	356.00	43.02	380.81	34.46
	**Asp**	2864.47	337.29	946.52	209.19	305.70	55.71	194.76	31.87
	**Pro**	9.30	0.03	19.38	2.76	41.04	2.81	87.69	8.59
	**Thr**	66.99	16.69	118.60	21.41	97.36	21.82	219.29	41.88
	**Frc**	2750.99	283.00	7866.80	1151.60	8942.77	1980.82	1968.79	272.00
	**Glc**	2032.26	398.69	7621.39	1122.87	10985.32	1901.39	2500.83	450.86
	**myo-ino**	544.71	63.91	688.14	59.51	876.34	113.53	405.86	47.17
	**Gltl**	20.52	4.57	51.40	12.30	66.74	7.77	76.69	14.51
	**Suc**	66592.66	3403.44	65764.01	3366.98	67971.33	3876.21	62139.28	3761.55
	**Mann**	11.27	1.30	14.56	2.27	27.99	4.12	33.43	4.55
	**Tre**	36.02	4.55	33.66	5.14	33.93	4.69	28.67	1.83
	**Raff**	349.57	41.68	671.74	97.45	1295.21	287.40	3107.40	634.71

	**Mal**	13.32	1.67	16.39	2.50	20.44	2.53	25.68	3.02
	**Succ**	94.47	11.09	78.10	11.45	53.32	8.70	37.86	3.20
	**Cit**	456.34	113.19	567.66	130.17	556.78	127.67	506.03	135.56
	**Oxal**	810.26	263.94	497.69	172.02	591.41	165.17	478.93	109.36
	**Glyc**	513.04	44.96	369.95	50.80	341.26	45.12	271.85	32.05
	**Mala**	1009.00	253.01	986.52	211.57	1290.18	306.92	1120.56	373.55
	**Acon**	132.06	27.50	111.12	19.30	132.37	54.74	73.05	11.21
	**Quin**	166.62	29.77	77.38	16.90	157.41	38.02	68.51	11.47
	**Shik**	233.00	37.45	171.02	18.69	264.70	48.89	189.96	32.32
	**Put**	13.30	2.24	17.51	2.44	27.92	3.83	35.20	3.72
**TQNG**	**Val**	21.54	5.09	37.20	4.27	91.94	8.84	105.20	6.14
	**Phe**	9.18	0.03	47.61	17.37	59.73	9.80	73.82	15.21
	**Ser**	169.12	23.43	352.81	57.94	488.09	41.65	270.78	35.14
	**Gln**	2449.03	209.38	7514.02	1202.56	5079.52	557.46	6296.48	1252.83
	**Ala**	609.68	81.50	506.94	37.41	368.27	24.21	412.54	41.75
	**Asp**	1660.68	415.37	323.24	46.52	302.59	37.56	156.04	26.24
	**Pro**	10.19	1.04	13.36	2.27	32.48	4.70	50.12	6.97
	**Thr**	36.93	8.20	193.91	50.56	247.60	44.60	236.63	47.32
	**Frc**	1836.74	348.84	2553.46	457.85	6379.89	480.83	3449.59	420.27
	**Glc**	1502.21	356.54	2829.20	530.50	8026.53	617.01	3871.60	361.28
	**myo-ino**	277.80	32.78	322.79	33.71	734.00	68.59	454.96	44.60
	**Gltl**	14.30	1.62	36.02	9.63	64.82	13.51	70.41	8.27
	**Suc**	66725.58	2422.03	66522.28	3355.69	64749.16	4479.54	68542.93	4247.13
	**Mann**	11.14	1.29	11.23	1.34	14.38	1.60	22.70	3.78
	**Tre**	29.69	3.30	19.53	2.43	25.73	4.60	24.51	2.12
	**Raff**	222.51	24.31	229.89	29.70	626.97	142.49	2876.76	669.96
	**Mal**	9.21	0.04	19.61	2.42	24.68	3.45	20.56	3.01
	**Succ**	62.48	5.26	46.13	5.11	53.27	8.06	35.73	4.11

	**Cit**	698.68	161.46	423.19	70.22	543.30	128.91	454.13	75.60
	**Oxal**	164.70	42.64	593.12	186.52	656.58	140.39	692.29	168.70
	**Glyc**	445.00	48.06	371.70	48.59	329.38	81.52	192.02	31.40
	**Mala**	599.50	138.04	1097.61	310.63	474.43	111.58	973.58	279.99
	**Acon**	72.15	10.30	93.47	10.88	128.76	37.99	82.79	10.23
	**Quin**	156.75	52.62	32.35	3.47	99.77	34.36	71.42	29.11
	**Shik**	149.42	15.67	124.32	13.24	188.06	39.42	298.50	52.31
	**Put**	11.23	1.32	13.24	1.58	15.39	1.53	26.89	2.47

Table S5B. Summary of C, I, C x I of metabolites. *p*-value were calculated for each effect using seven cultivars (C), four irrigations (I), and their interaction (C x I). ** and ns indicate $p < 0.01$ and $p > 0.01$, respectively.

Metabolite	C	I	C x I
**Ala**	**	**	**
**Asp**	**	**	**
**Leu**	**	**	**
**Pro**	**	**	**
**Val**	**	**	**
**Ser**	*	**	**
**Frc**	**	**	**
**Glc**	**	**	**
**Mal**	**	**	**
**myo-ino**	**	**	**
**Quin**	**	**	**
**Shik**	**	**	**
**Succ**	**	**	**
**Gln**	ns	**	**
**Glyc**	ns	**	*
**Phe**	ns	**	*
**Mala**	ns	*	**
**Raff**	ns	**	**
**Tre**	*	ns	**
**Acon**	ns	ns	**

**Cit**	ns	ns	**
**Suc**	ns	ns	*
**Gly**	ns	**	ns
**Ile**	ns	**	ns
**Oxal**	ns	*	ns
**Gltl**	*	**	ns
**Mann**	**	**	ns
**Put**	**	**	ns
**Thr**	*	**	ns

Table S6. Summary of correlation between grain yield and 5 physiological traits. Correlation coefficients and *p*-value were calculated by using data from all four irrigation levels to include the effects of water stress. ** indicates $p < 0.01$.

Var 1	Var 2	Correlation coefficient	*p*-value
Yield	A	0.43	**
	g_s	0.53	**
	WUE (A/g_s)	-0.24	**
	Leaf Tm	-0.17	**
	SPAD	-0.27	**

Table S7. Summary of visible stress symptoms in response to IRRI_1 to IRRI_4 were recorded on the same date when metabolic samples and physiological data (84 DOE) were collected in 2016.

Yield performance	Cultivar	IRRI_1	IRRI_2	IRRI3	IRRI_4
High response	**TQNG**	No symptoms	No symptoms	No symptoms	No symptoms
	**PI77**	No symptoms	No symptoms	No symptoms	No symptoms
Intermediate response	**LGRU**	No symptoms	No symptoms	No symptoms	Wilted, leaf curling
	**LMNT**	No symptoms	No symptoms	No symptoms	Wilted, leaf curling
Non- or low response	**KBNT**	No symptoms	No symptoms	No symptoms	Wilted, leaf curling
	**SABR**	No symptoms	No symptoms	No symptoms	Wilted, leaf curling
	**FRCS**	No symptoms	No symptoms	No symptoms	No symptoms

Table S8. Bivariate fit of metabolites as a function of % volumetric water content (VWC) as determined by irrigation treatment. Polynomial fits of high, intermediate, and low response cultivars were calculated respectively. H, I, L represent high, intermediate, and low response cultivars. **, * and ns indicate $p < 0.01$, $p < 0.05$ and $p > 0.05$, respectively.

	IRRI. Treat	Metabolites	Yield response category	RSquare Adj	Prob > F		Intercept (Prob>|t|)		IRRI. Treat (Prob>|t|)		(IRRI. Treat-25.1084)^2 (Prob>|t|)	
Cluster 1	% VWC	Frc	H	0.22	7.78E-05	**	5.23E-12	**	0.000273	**	0.226394198	ns
			I	0.42	1.97E-09	**	2.8E-10	**	0.767263	ns	1.11348E-09	**
			L	0.42	2.28E-13	**	9.14E-13	**	0.166561	ns	7.32196E-14	**
	% VWC	Glc	H	0.25	1.58E-05	**	1.81E-12	**	0.000268	**	0.063695663	ns
			I	0.39	1.67E-08	**	1.2E-09	**	0.902967	ns	1.49332E-08	**
			L	0.33	7.14E-10	**	3.39E-09	**	0.593863	ns	4.30194E-10	**
	% VWC	myo_ino	H	0.29	3.21E-06	**	2.51E-15	**	1.6E-05	**	0.205069504	ns
			I	0.19	0.000265	**	5.5E-12	**	0.583292	ns	0.000262993	**
			L	0.23	4.56E-07	**	2.74E-13	**	0.156212	ns	8.49195E-08	**
	% VWC	Acon	H	0.06	0.047557	*	4.92E-07	**	0.019272	*	0.956049211	ns
			I	-0.03	0.896684	ns	0.008473	**	0.665935	ns	0.984488018	ns
			L	0.06	0.012902	*	0.172911	ns	0.005409	**	0.926705213	ns
	% VWC	Tre	H	0.02	0.169578	ns	8.03E-08	**	0.076006	ns	0.23591081	ns
			I	0.02	0.221332	ns	0.003407	**	0.101841	ns	0.99232508	ns
			L	0.04	0.03847	*	1.3E-08	**	0.083946	ns	0.017304063	*
Cluster 2	% VWC	Ala	H	0.44	7.5E-10	**	0.495443	ns	1.86E-09	**	0.507436696	ns
			I	0.55	4.09E-13	**	0.06441	ns	3.4E-12	**	0.248021391	ns
			L	0.51	4.11E-17	**	0.032211	*	3.35E-16	**	0.720581818	ns
	% VWC	Asp	H	0.58	3.29E-14	**	1.95E-07	**	1.71E-10	**	0.000319085	**

			I	0.75	5.28E-22	**	2.2E-13	**	3.13E-15	**	4.4111E-09	**
			L	0.64	1.35E-23	**	9.97E-13	**	3.76E-18	**	8.86342E-05	**
	% VWC	Succ	H	0.13	0.002552	**	0.002731	**	0.001216	**	0.934578064	ns
			I	0.10	0.011786	*	2.29E-06	**	0.079587	ns	0.004180568	**
			L	0.25	1.41E-07	**	0.117438	ns	2.2E-08	**	0.013816656	*
	% VWC	Glyc	H	0.17	0.000719	**	0.032305	*	0.000231	**	0.028110616	*
			I	0.01	0.251053	ns	7.77E-06	**	0.526012	ns	0.097988731	ns
			L	0.12	0.000429	**	0.004182	**	0.000111	**	0.033384457	*
	% VWC	Suc	H	-0.01	0.616676	ns	3.59E-23	**	0.926646	ns	0.373898191	ns
			I	0.01	0.311873	ns	1.37E-23	**	0.376776	ns	0.143009782	ns
			L	0.05	0.029774	*	3.05E-33	**	0.216916	ns	0.008553823	**
	% VWC	Quin	H	0.17	0.000604	**	0.159313	ns	0.015485	*	0.03072927	*
			I	0.26	1.4E-05	**	0.022188	*	0.001341	**	0.012685533	*
			L	0.00	0.334438	ns	0.20731	ns	0.143479	ns	0.47284016	ns
	% VWC	Shik	H	-0.01	0.447283	ns	4.75E-05	**	0.769145	ns	0.208867967	ns
			I	0.04	0.086346	ns	2.83E-06	**	0.368869	ns	0.105009159	ns
			L	0.02	0.103725	ns	2.8E-07	**	0.635934	ns	0.076974001	ns
Cluster 3	% VWC	Oxal	H	0.02	0.189189	ns	6.33E-05	**	0.156076	ns	0.53871716	ns
			I	0.10	0.009727	**	0.000994	**	0.954124	ns	0.003901113	**
			L	-0.01	0.783397	ns	0.002269	**	0.984921	ns	0.511396522	ns
	% VWC	Ser	H	0.21	0.000106	**	1.78E-13	**	0.02599	*	0.003933901	**
			I	0.44	7.76E-10	**	1.9E-21	**	4.53E-09	**	0.263230398	ns
			L	0.16	3.85E-05	**	9.65E-17	**	0.001983	**	0.034760545	*
	% VWC	Gln	H	0.19	0.000226	**	3.19E-11	**	0.292582	ns	0.00053809	**

			I	0.06	0.046984	*	2.95E-10	**	0.03648	*	0.572363212	ns
			L	0.13	0.000292	**	7.19E-16	**	0.005985	**	0.060160695	ns
	% VWC	Mal	H	-0.01	0.460714	ns	5.84E-05	**	0.570085	ns	0.218797347	ns
			I	0.33	4.51E-07	**	5.4E-16	**	7.2E-08	**	0.035840911	*
			L	0.27	2.94E-08	**	2.51E-19	**	6.61E-09	**	0.001243058	**
	% VWC	Cit	H	0.29	3.16E-06	**	0.13907	ns	1.83E-05	**	0.181416141	ns
			I	-0.01	0.552456	ns	0.019907	*	0.28353	ns	0.60387314	ns
			L	0.00	0.434252	ns	8.13E-05	**	0.535196	ns	0.404847082	ns
	% VWC	Mala	H	0.05	0.072476	ns	0.476919	ns	0.022742	*	0.53144537	ns
			I	0.00	0.39965	ns	0.041675	*	0.217512	ns	0.913673094	ns
			L	-0.01	0.591566	ns	1.88E-05	**	0.377768	ns	0.869093575	ns
Cluster 4	% VWC	Val	H	0.66	3.21E-17	**	1.6E-25	**	5.14E-18	**	0.011118307	*
			I	0.76	1.35E-22	**	4.1E-28	**	2.22E-23	**	3.3385E-09	**
			L	0.66	5.11E-25	**	1.2E-35	**	5.67E-26	**	4.24188E-05	**
	% VWC	Pro	H	0.58	2.68E-14	**	2.22E-19	**	4.65E-15	**	2.11418E-05	**
			I	0.76	8.53E-23	**	1.58E-28	**	1.27E-23	**	6.6528E-09	**
			L	0.76	3.94E-33	**	6.35E-42	**	3.78E-34	**	4.86579E-11	**
	% VWC	Put	H	0.49	3.28E-11	**	3.94E-20	**	4.47E-12	**	0.002905115	**
			I	0.47	1.33E-10	**	1.96E-19	**	2.04E-11	**	0.04545614	*
			L	0.51	5.23E-17	**	2E-33	**	6.45E-18	**	0.002434947	**
	% VWC	Raff	H	0.52	4.6E-12	**	5.17E-14	**	1.67E-12	**	8.32829E-06	**
			I	0.51	6.88E-12	**	8.57E-14	**	3.07E-12	**	5.3091E-06	**
			L	0.62	2.68E-22	**	8.97E-29	**	2.9E-23	**	6.61417E-07	**
	% VWC	Ile	H	0.75	9.88E-22	**	5.78E-29	**	1.1E-22	**	2.12464E-06	**

			I	0.69	9.96E-19	**	9.76E-25	**	1.2E-19	**	7.95756E-06	**
			L	0.59	7.88E-21	**	1.05E-28	**	9.03E-22	**	1.28145E-06	**
			H	0.67	1.49E-17	**	1.48E-24	**	1.79E-18	**	3.53604E-05	**
	% VWC	Leu	I	0.70	4.43E-19	**	4.3E-24	**	8.73E-20	**	6.05624E-08	**
			L	0.60	2.5E-21	**	1.02E-29	**	2.59E-22	**	3.79591E-06	**
			H	0.33	4.49E-07	**	7.97E-14	**	7.71E-08	**	0.123341665	ns
	% VWC	Gly	I	0.36	8.67E-08	**	9.89E-16	**	1.56E-08	**	0.145404235	ns
			L	0.55	6.24E-19	**	5.67E-35	**	9.02E-20	**	0.00373693	**
			H	0.47	1.08E-10	**	1.1E-16	**	1.72E-11	**	0.056278786	ns
	% VWC	Phe	I	0.37	4.53E-08	**	4.9E-11	**	8.04E-09	**	0.005345737	**
			L	0.49	4.05E-16	**	3.79E-22	**	4.6E-17	**	0.000133516	**
			H	0.26	1.44E-05	**	6.04E-12	**	9.09E-06	**	0.905256471	ns
	% VWC	Thr	I	0.31	9.97E-07	**	3.84E-13	**	1.64E-07	**	0.086081168	ns
			L	0.11	0.001294	**	1.82E-11	**	0.000716	**	0.945510317	ns
			H	0.31	1.22E-06	**	3.36E-12	**	2.18E-07	**	0.15142417	ns
	% VWC	Gltl	I	0.37	3.4E-08	**	2.9E-13	**	6.53E-09	**	0.0032021	**
			L	0.17	3.04E-05	**	1.32E-14	**	5.25E-05	**	0.543741527	ns
			H	0.34	2.28E-07	**	1.43E-14	**	4.55E-08	**	0.005578526	**
	% VWC	Mann	I	0.44	7.64E-10	**	4.71E-20	**	1.82E-10	**	0.236516341	ns
			L	0.52	3.03E-17	**	1.19E-28	**	3.29E-18	**	0.000117546	**

Table S9. Summary of the % yield, g_s, and A reduction at each irrigation level compared to IRRI_1 in 2016.

	**Yield**	g_s	**A**

	IRRI 1 (g)	% change IRRI 1 - IRRI 2	% change IRRI 1 - IRRI 3	% change IRRI 1 - IRRI 4	IRRI 1	% change IRRI 1 - IRRI 2	% change IRRI 1 - IRRI 3	% change IRRI 1 - IRRI 4	IRRI 1	% change IRRI 1 - IRRI 2	% change IRRI 1 - IRRI 3	% change IRRI 1 - IRRI 4
**PI77**	47.0	-23	-44	-56	0.86	-51	-63	-83	14.2	-1	-10	-40
**TQNG**	47.0	19	-22	-39	0.63	-6	-59	-90	16.6	0	-28	-60
**FRCS**	37.7	-8	22	-22	0.38	3	-42	-71	15.5	-9	-17	-60
**LGRU**	26.5	-29	-49	-65	0.44	-25	-55	-95	15.2	-5	-13	-71
**SABR**	24.5	-1	-5	-14	0.29	14	-10	-59	13.4	5	-2	-60
**LMNT**	17.3	11	-3	-77	0.38	-29	-61	-74	15.2	-14	-43	-49
**KBNT**	14.2	14	36	6	0.23	4	-13	-78	11.3	1	-8	-57

Table S10. Means and SE of heading dates of 7 rice cultivars under 4 different irrigation levels (IRRI_1 to 4).

Year	Cultivars	IRRI. Treat	Days to Heading	
			Mean	SE
2016	FRCS	1	95	0.30
		2	94.6	0.63
		3	95.6	1.72
		4	97.7	1.45
	KBNT	1	104.5	1.76
		2	102.55	0.40
		3	100.3	0.72
		4	105.3	0.69
	LGRU	1	107.1	1.10
		2	105	0.55
		3	108.7	2.22
		4	108.6	2.23
	LMNT	1	100.8	1.08
		2	103.6	0.17
		3	103.3	0.92
		4	108	0.40
	PI77	1	95	0.61
		2	104.5	1.73
		3	106.6	1.23

		4	107.3	0.88
	SABR	1	96.8	1.01
		2	105	2.12
		3	103.8	0.72
		4	102	0.81
	TQNG	1	103.8	0.72
		2	102.8	1.33
		3	107	1.33
		4	109	0
